# Fungus of the Eye

**Published:** 1829-02

**Authors:** 


### FUNGUS OF THE EYE.

*Case of Fungus of the Globe of the Eye.* By W. Twining, Esq.

The following case of tumor growing from the globe of the eye, which came under my care, when acting as oculist during Mr. Egerton's absence last year, may be worthy of record, as an example that some tumors of very formidable appearance, and productive of severe pain, have nothing in their nature which is malignant, or likely to communicate disease to the contiguous parts; and that such diseases may be extirpated with success.

A healthy, but rather slight-made Hindoo, about fifty years of age, applied at the Eye Infirmary, Calcutta, February 17, 1826, having a fungus that protruded from between the right eyelids, larger than an egg: it was of a red colour, hard, and its surface irregular or granular, like a firm cauliflower, and, when handled or pressed, there was little bleeding, but always a puriform discharge.

The tumor appeared to grow from the whole of the eyeball: it was moveable, and its base was firmly girt by the eyelids, but not attached to them; in fact, there was no very strong attachment apparent, except at the lachrymal gland. It filled the whole front of the orbit, and, protruding as above stated, was not only a hideous deformity, but was productive of severe pain in the orbit and head. The weight and pressure of the protruded portion of the tumor which rested on the cheek, had there caused ulceration of the skin. The disease had been fourteen months in arriving at this state, and was preceded by inflammation, which the man said arose from a particle of straw blown into his eye by the wind.

On the 5th March, I extirpated the disease with a common scalpel. The base of the tumor was so firmly girt by the aperture of the eyelids, that an incision was first requisite from the outer corner of the eye towards the temple, so as to give more room for the operation, which was then accomplished in less than a minute by a few strokes of the knife. Two arteries bled freely, and were secured by ligatures. A piece of lint was placed over the closed lids, and over that a sponge retained by a bandage.

There was no unfavorable symptom afterwards, and the man was discharged cured at the end of the month. This man presented himself at the Eye Infirmary a few days ago, (February 1827,) remaining quite well; and there does not appear any tendency to a return of the disease.

After the operation, on making an incision across the tumor, it was found of a firm granular texture throughout; and in its centre the remains of the eye were very evident. The whole globe of the eye, with a portion of the optic nerve, were removed; as may be seen on inspecting the preparation, which is now at the Eye Infirmary.

On maceration in spirit, the tumor became of a white colour, and shrunk to about half its original size; but still the shape of the eyeball is distinctly marked by the pigment of the choroid.

---

